# Comparative transcriptome profile of the leaf elongation zone of wild barley (*Hordeum spontaneum*) *eibi1* mutant and its isogenic wild type

**DOI:** 10.1590/1678-4685-GMB-2016-0321

**Published:** 2017-10-23

**Authors:** Qin Zhou, Aidong Wang, Ruijun Duan, Jun Yan, Gang Zhao, Eviatar Nevo, Guoxiong Chen

**Affiliations:** 1 Northwest Institute of Eco-Environment and Resources, Chinese Academy of Sciences, Lanzhou, China; 2 University of Chinese Academy of Sciences, Beijing, China; 3 College of Eco-Environmental Engineering, Qinghai University, Xining, Qinghai, China; 4 School of Pharmacy and Bioengineering, Chengdu University, Chengdu, Sichuan. China; 5 Institute of Evolution, University of Haifa, Haifa Israel

**Keywords:** ABC transporter, defense genes, desiccation tolerance, plant cuticle, RNA-Seq

## Abstract

The naturally occurring wild barley mutant *eibi1/hvabcg31* suffers from severe water loss due to the permeable leaf cuticle. *Eibi1/HvABCG31* encodes a full ATP-binding cassette (ABC) transporter, HvABCG31, playing a role in cutin deposition in the elongation zone of growing barley leaves. The *eibi1* allele has pleiotropic effects on the appearance of leaves, plant stature, fertility, spike and grain size, and rate of germination. Comparative transcriptome profile of the leaf elongation zone of the *eibi1* mutant as well as its isogenic wild type showed that various pathogenesis-related genes were up-regulated in the *eibi1* mutant. The known cuticle-related genes that we analyzed did not show significant expression difference between the mutant and wild type. These results suggest that the pleiotropic effects may be a compensatory consequence of the activation of defense genes in the *eibi1* mutation. Furthermore, we were able to find the mutation of the *eibi1/hvabcg31* allele by comparing transcript sequences, which indicated that the RNA-Seq is useful not only for researches on general molecular mechanism but also for the identification of possible mutant genes.

## Introduction

The cuticle covers aerial plant organs and acts as an effective barrier against pests and pathogens (Reina-Pinto and Yephremov, 2009; Metraux *et al.*, 2014). The cuticle mainly consists of wax and cutin. Cutin is made up of polyesters whose monomer composition is mainly C16 and C18 ω-hydroxylated fatty acid, and is typically modified by hydroxy- or epoxy groups in mid-chain positions (Yeats and Rose, 2013). Moreover, cutin contains glycerol and a small amount of phenolic compounds (Nawrath, 2006; Yeats and Rose, 2013).

A spontaneous cuticle mutant (*eibi1*) from wild barley (*Hordeum spontaneum*) genotype 23-19 has been cloned via a map-based approach (Chen *et al.*, 2009, 2011b,). The *eibi1* mutant suffers from severe water loss related to a reduced cuticle thickness and a decreased amount of cutin monomers, and shows twisted leaves, dwarf plant, low fertility, reduced spike and grain size, and germination delay. The gene, named as *Eibi1/HvABCG31* is mapped to a pericentromeric region on chromosome 3H, where a mutation in an ABCG31 transporter gene is associated with the *eibi1* mutant phenotype. This PDR (pleiotropic drug resistance) transporter family, as well as its homologs in Arabidopsis and rice, AtABCG32 and OsABCG31, plays an important role in cutin deposition during the development of a functional cuticle (Bessire *et al.*, 2011; Chen *et al.*, 2011a; Garroum *et al.*, 2016). Analysis of *Eibi1* gene expression shows abundant transcripts in the elongation zone (EZ) but only traces in non-elongation zones (NEZ) and emerged blade (EmBL) of a growing leaf, and none in the mature root (Chen *et al.*, 2011b). Cutin but not wax deposition occurs mostly in the EZ (Richardson *et al.*, 2007). Cutin deposition is already established by the time the EZ is formed, and the defect of the *eibi1* mutant cuticle is apparent in the EZ (Chen *et al.*, 2011b). However, gene transcription in *eibi1* EZ is poorly understood.

A comparative transcriptomic analysis of the second leaves of near-isogenic *eibi1* and the wild type lines has been conducted using the 22-k Barley1 Affymetrix microarray and the results revealed a pleiotropic effect of *HvABCG31* gene on cuticle biogenesis and stress responsive pathways (Yang *et al.*, 2013). In the present study, we conducted the transcriptome analysis (RNA-Seq) in the EZ of the third leaf of *eibi1* mutant as well as its wild type, 23-19, with four purposes: (a) to reveal the transcriptome of the wild barley leaf EZ; (b) to test if RNA-Seq can be used for *eibi1* mutation identification; (c) to test if the mutation affects the expression of other cuticle-related genes; and (d) to identify the major effects of *eibi1* mutation on the other genes at the expression level.

## Materials and Methods

### Plant material

The wild barley accession 23-19 was selected from the Wadi Qilt population maintained at the Gene Bank of the Institute of Evolution, University of Haifa, Israel. The *eibi1* mutant arose from 23-19 (Chen *et al.*, 2004). Caryopses of the *eibi1* mutant and wild type 23-19 were sown in 2.5-L pots filled with commercial compost, and the plants were grown in an incubator at 65% relative humidity, 22/16 °C (12-hour day/night cycle) under fluorescent light. The EZ of leaf three was sampled from a seedling at three-leaf stage. Six independent replicate samples were mixed for RNA extraction for both mutant and wild type. The EZ in this research is the region within 25 mm from leaf three insertion point.

### RNA extraction, quality determination and validation of RNA-Seq by qRT-PCR

Total RNA of mutant or wild type was isolated using RNA kit (Tiangen, Beijing, China) following manufacturer’s instructions. The concentration and quality of RNA were verified by absorption 260/280 nm ratio between 1.8 and 2.0 using Nanodrop2000 spectrophotometer (Thermo Scientific, USA). RNA quality was checked on a Bioanalyzer 2100 (Agilent, Santa Clara, CA, USA) and RNA integrity number (PRIN) values were greater than 8.6 for mutant and wild type.

Total RNA from 23-19 wild type and *eibi1* mutant were treated with DNase and reverse-transcribed using a reverse transcription kit (RR047A) (Takara Biomedical Technology, Beijing, China). Quantitative RT-PCR was performed in an Agilent Stratagene MX3000P (Agilent) using Power SYBR green chemistry (RR820A) (Takara). All qRT-PCR reactions were performed in triplicate following the reaction conditions: 95 °C for 10 min followed by 40 cycles of 95 °C for 10 s and 60 °C for 31 s, and the results were analyzed with the relative quantification system based on the 2^-ΔΔCt^ method. Actin primers were 5’-AAGTACAGTGTCTGGATTGGAGGG-3’ (sense) and 5’-TCGCAACTTAGAAGCACTTCCG-3’ (antisense).

### Illumina cDNA library preparation and sequencing

For cDNA synthesis and sequencing, 20 μg of total RNA was used, at a concentration of ≥ 400 ng/μL, one technical replicate for both mutant and wild type. The cDNA libraries were constructed using an mRNA-Seq assay with a fragment length range of 200 bp (± 25 bp). The libraries were sequenced for paired-end reads of 90 bp using Illumina HiSeq 2000 platform (Illumina, San Diego, CA, USA) at the Beijing Genomics Institute (Shenzhen, China).

### *De novo* assembly and assessment

Reads from each library were assembled separately. Raw reads that contained adapters and unknown or low-quality bases were discarded by (1) finding the reads containing adapters and to removing the adapters; (2) removing reads with more than 5% unknown nucleotides; and (3) removing low-quality reads using in-house Perl scripts. Transcriptome *de novo* assembly was conducted with Trinity (version 20130225) (Grabherr *et al.*, 2011). Trinity-assembled reads were grouped to form longer fragments without N, named as contigs. Then, in order to detect contigs in the same transcripts and the distances between them, we mapped reads back to contigs. Next, Trinity connected the contigs using N to represent unknown sequences between each two contigs, and then scaffolds were made. Finally, we got sequences without Ns that could not be extended, which were defined as Unigenes. TGICL (TIGR Gene Indices clustering tools) (Pertea *et al.*, 2003) was then used to assemble all the unigenes from different samples to form a single set of non-redundant unigenes. Single-nucleotide polymorphism (SNP) profiling to compare 23-19 wildtype and *eibi1* mutant was done using SOAPsnp (Short Oligonucleotide Alignment Program for SNP detection) (http://soap.genomics.org.cn) (Li *et al.*, 2009) after the Unigene sequence was assembled.

### Functional annotation

We identified possible protein coding regions within the assembled Unigenes using the TransDecoder program implemented in the Trinity software and then analyzed protein sequences for Pfam matches to obtain Pfam annotation and alignments (Finn *et al.*, 2016) in Pfam 31.0 database (http://pfam.xfam.org/) (Schaeffer *et al.*, 2017). The pfam2go mapping (http://www.geneontology.org/external2go/pfam2go) was used to map Pfam annotation to GO terms for All-Unigenes.

### Analysis of differentially expressed genes

To identify differentially expressed genes (DEG), we examined counts (the number of overlapping reads for each coding region) in the *eibi1* and 23-19 libraries by empirical analysis of DEG in EdgeR, which is part of the Bioconductor project (Robinson *et al.*, 2009; McCarthy *et al.*, 2012). Gene expression levels were measured in the RNA-Seq analyses as numbers of reads per kilobase of exon region in a given gene per million mapped reads (RPKM), a normalized measure of exonic read density that allows transcript levels to be compared both within and between samples (Mortazavi *et al.*, 2008). The results of all statistical tests were corrected for multiple testing with the Benjamini–Hochberg false discovery rate (FDR). In our analysis, we chose those with FDR ≤ 0.05 and ratio larger than 2. A gene is defined as absent when its RPKM value is zero.

BinGO (Cytoscape 3.3.0) (Maere *et al.*, 2005) was used for Gene Ontology (GO) functional classification. The calculated p-value was submitted to the Bonferroni correction, using the corrected p-value ≤ 0.05 as a threshold. GO terms fulfilling this condition were defined as significantly enriched GO terms in DEGs.

### Identification of EST polymorphisms

The sequences of expressed sequence tags (EST) in the Morex *eibi1* (155F_2_) genetic map were retrieved from the NCBI nucleotide database (http://www.ncbi.nlm.nih.gov/nucleotide/). Corresponding *eibi1* Unigenes were identified by local blastn against Morex contigs that were found by blastn with EST sequences as queries against the Morex sequence database: assembly3_WGSMorex_renamed_blastable_POPSEQ.fasta (http://webblast.ipk-gatersleben.de/barley). EST polymorphisms between *eibi1* and Morex were identified by the comparison between *eibi1* Unigenes and Morex contigs.

## Results

### The transcriptome of the barley leaf elongation zone

**Figure 1:**
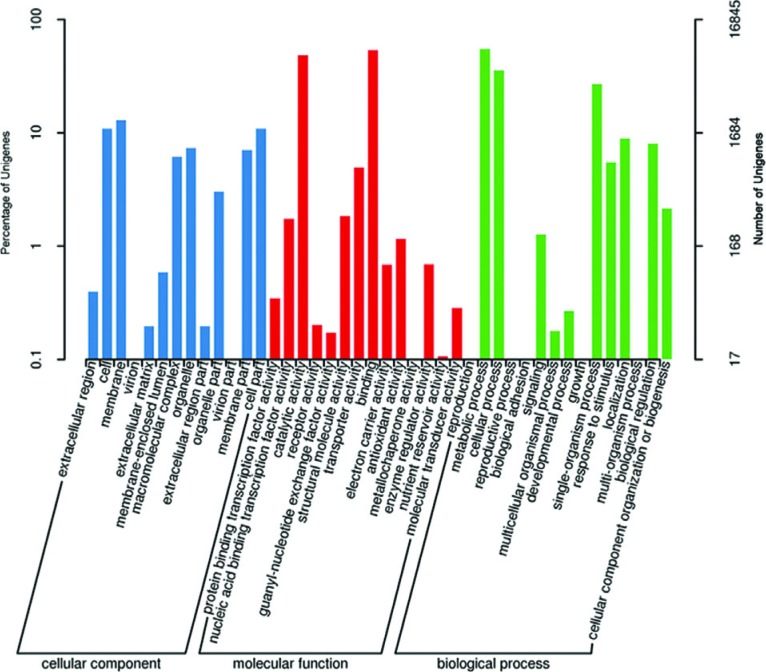
GO classification of annotated Unigenes.

About 54 million and 51 million clean reads were generated from the 23-19 and *eibi1* cDNA libraries, respectively (Table 1). A total of 78,799 Unigenes with the average length of 645bp were generated. The size distribution of the assembly is shown in Table S1 available as Supplementary Data. Distribution of unique-mapped reads of the assembled unigenes of 23-19 and *eibi1* are shown in Table S2. The entire Unigene sets were then annotated with Pfam database. Among the 78,799 unique sequences, 16,845 Unigenes were annotated using the public databases Pfam with GO terms. BinGO tool was used to perform the enrichment analysis. In the biological process category, 10,077 Unigenes (60%) were involved in cellular and metabolic process. In the cell component category, more than 3,721 Unigenes (22%) played roles in cell, membrane and organelle. In the molecular function category, about 14,717 Unigenes (87%) were involved in metabolic and cellular processes (Table S3, Figure 1).

**Table 1:** Read number and statistics based on the RNA-Seq data in the wild type and mutant.

Samples	Total Reads	Total Nucleotides (nt)	Q20 percentage	N percentage	GC percentage*
23-19	54,821,138	4,933,902,420	96.25%	0.00%	53.69%
*eibi1*	51,177,934	4,606,014,060	96.41%	0.00%	53.13%

Total Reads and Total Nucleotides are clean reads and clean nucleotides; Q20 percentage is the proportion of nucleotides with quality values larger than 20; N percentage is the proportion of unknown nucleotides in clean reads; GC percentage is the proportion of guanidine and cytosine nucleotides among total nucleotides.

### The identification of *eibi1* mutation via RNA-Seq

**Figure 2:**
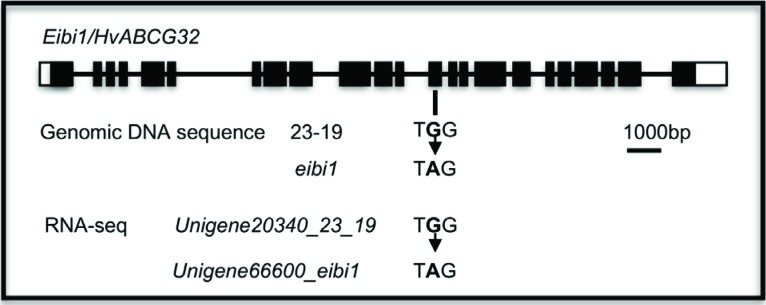
Identification of *eibi1* mutation by RNA-Seq. Unigene20340_23-19 and Unigene66600_*eibi1* were assembled from RNA-Seq of wild-type 23-19 and mutant *eibi1*, respectively. The SNPs are indicated by arrows.

We compared the sequences between mutant and its isogenic wild type and found 5 unigenes with SNPs (Table S4). Among those, Unigene19902_All (the combination of Unigene20340_23-19 and Unigene66600_*eibil*) is an ABCG transporter gene which may be involved in cutin secretion (Chen *et al.*, 2009, 2011b). Thus, Unigene19902_All is likely the candidate gene for *Eibi1*. Actually, Unigene19902_All was the corresponding sequence of *Eibi1*; it exhibited a SNP exactly as found in our previous study (Figure 2) (Chen *et al.*, 2011b). The depth of the Unigene19902_All was 63 in *eibi1* and 320 in wild type. The coverage of this Unigene was 97% and 50% in *eibi1* and 23-19, respectively. The base substitution (G → A) at position 294 mapped 56 and 255 reads of the assembled Unigenes of *ebi1* and 23-19, respectively (Table S4). This result suggests that the RNA-Seq data can be used for the cloning of a mutant gene.

**Figure 3:**
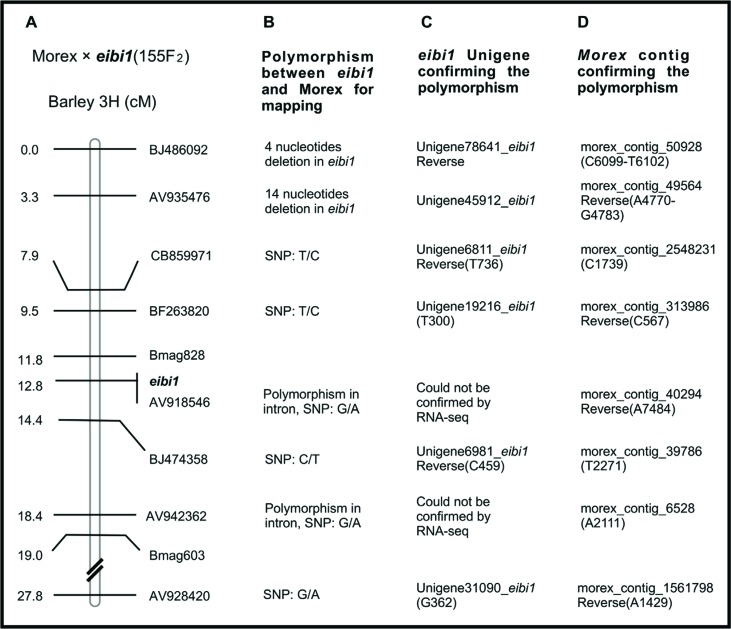
An example of marker development by RNA-Seq. (A) *eibi1* genetic map. Bmag828 and Bmag603 are SSR markers. The others are EST markers. A total of 155 F_2_ individuals derived from the cross of Morex *eibi1* were used for the genetic mapping. The numbers at the left side indicate genetic distances in centimorgan (cM). (B) Corresponding marker polymorphism between parental line Morex and *eibi1*. The polymorphisms were identified by PCR sequencing against parental genomic DNA. (C) Corresponding *eibi1* Unigenes (derived from RNA-Seq) for the EST markers. Inside the parenthesis is a nucleotide followed by its position in the corresponding Unigene sequence. (D) Corresponding Morex contigs for the EST markers were retrieved from the database: assembly3_WGSMorex_renamed_blastable_POPSEQ.fasta (http://webblast.ipk-gatersleben.de/barley). Inside the parenthesis is a nucleotide followed by its position in the corresponding contig sequence.

Because a mutant gene was not expressed in the tissue sampled for the RNA-Seq, we could not find the corresponding mutation. However, RNA-Seq data can be used to find polymorphisms in Unigenes between parental lines of an F_2_ population for genetic mapping of the mutant. As an example, in Morex *eibi1* (155F_2_) genetic map (Figure 3) (Chen *et al.*, 2009), six out of eight EST-derived markers were confirmed by *eibi1* Unigenes and Morex contigs (Table S5), indicating that the results of the present RNA-Seq study were reliable.

### Differentially expressed genes found in *eibi1* mutant

**Figure 4:**
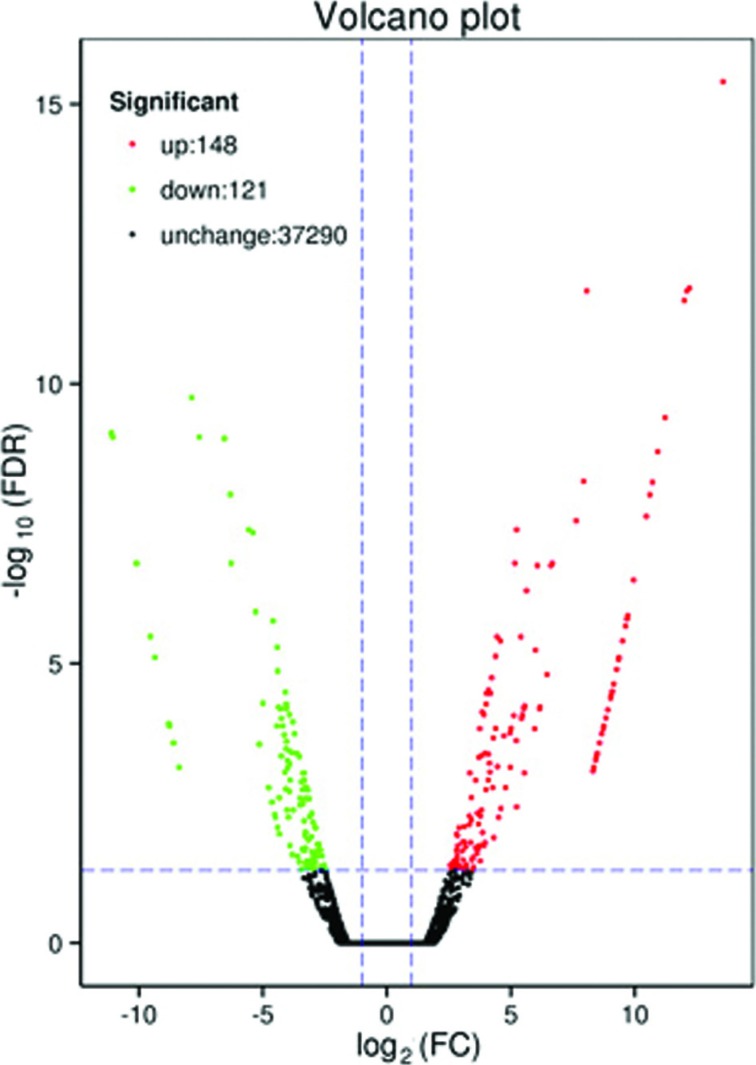
Expression level of DEGs of 23-19 and *eibi1* libraries*.* All Unigenes were examined for differences in their expression across the two samples. Red (up) and green (down) dots denote that the expression was significantly different (FDR ≤ 0.05, 2-fold difference) and the blank dots indicate no change between the expression levels in the two varieties.

About 269 Unigenes were identified as DEGs between *eibi1* and 23-19 samples (Table S6), 148 up-regulated and 121 down-regulated in the mutant (Figure 4). Only 110 DEGs were annotated in the Pfam with GO terms (Table S7), which covered only half of differentially expressed Unigenes. To explore DEG results more deeply, within the top 20 up- and down-regulated DEGs that failed Pfam2go mapping were analyzed (Table S8) via barley genome annotation (International Barley Genome Sequencing Consortium 2016) (Beier *et al.*, 2017).

In order to find out if cuticle-related genes were affected by *eibi1* mutation, we searched 75 known cuticle genes (Table S9) (Nawrath, 2006; Yeats and Rose, 2013) against the RNA-Seq data by BLAST and examined their expression level. We found 66 genes that had at least one homolog in the RNA-Seq data (E-value e < 0.00001). But no homolog showed significant differential expression between *eibi1* and its wild parental line 23-19.

### Validation of differentially expressed genes

**Figure 5:**
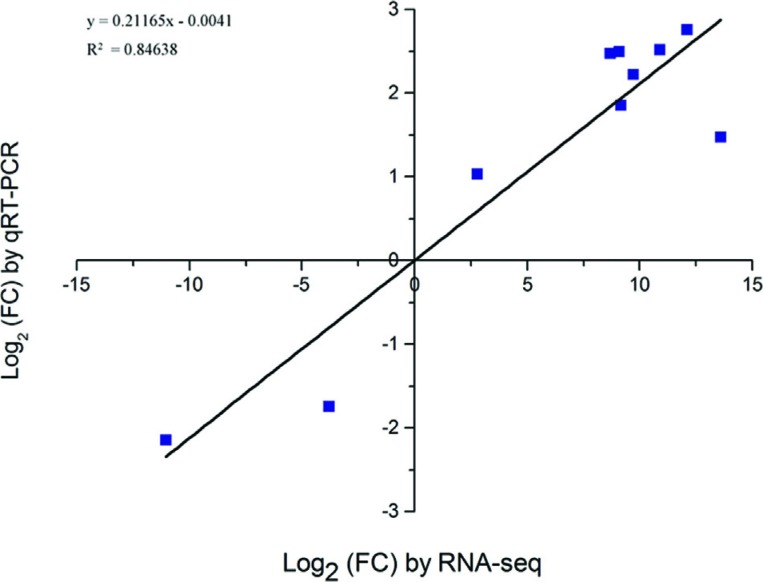
Comparison of log_2_ (FC) of 10 select transcripts using RNA-Seq and qRT-PCR. FC: fold change for *eibi1* vs 23-19.

To assess the accuracy of DEGs, a subset of 10 DEGs in *eibi1* and 23-19 were analyzed by quantitative real-time PCR (qRT-PCR). Their primers were designed based on barley sequences obtained from RNA-Seq, and are listed in Table S10. We tested their expression results obtained from qRT-PCR with those generated by the RNA-Seq, which were calculated based on the median of three repeats, and showed good consistency for all transcripts in both analyses, with a correlation coefficient R^2^ = 0.84638 (Figure 5). Hence, results of the DEG analysis are trustworthy, and its results are suitable for further investigation.

### Functional annotation of differentially expressed genes

As shown in Table 2, Unigene65566_All and Unigene55562_All, annotated as GDSL esterase/lipase, were significantly down-regulated in *eibi1*. Unigene21078_All encoding Lipase as well as Unigene57532_All and Unigene60434_All encoding glycerophosphodiester phosphodiesterase 1 was slightly down-regulated in mutant. Meanwhile, lipid-transfer protein (Unigene75732_All), lipoxygenase (Unigene72225_All) and fatty acid elongases (Unigene6358_All) were up-regulated in *eibi1*.

**Table 2:** Relative expression of lipid metabolic process related Unigenes.

Unigene ID	Related function	Log_2_ (*eibi1*_RPKM/23-19_RPKM)
Unigene65566_All	GDSL esterase/lipase	-11.1044
Unigene55562_All	GDSL esterase/lipase	-11.0571
Unigene21078_All	Lipase (class 3)	-3.27893
Unigene60434_All	Lipid metabolic process	-3.25452
Unigene57532_All	Lipid metabolic process	-2.52976
Unigene75732_All	Bifunctionalinhibitor/lipid-transfer protein	9.362752
Unigene72225_All	Lipoxygenase	3.441247
Unigene6358_All	FAE1/Type III polyketide synthase-like protein	2.723891

Column named log_2_ (*eibi1*_RPKM/23-19_RPKM) indicates the expression of Unigenes in *eibi1* compared to 23-19. Plus (omitted): up-regulated, minus: down regulated.

The most up-regulated DEGs were defense-related genes (Table 3), such as pathogenesis-related (PR) genes (Unigene_78697_All and Unigene_78238_All), *WIR1A* (*wheat induced resistance 1*) (Unigene66257_All) (Coram *et al.*, 2010), horcolin (*Hordeum vulgare* coleoptile lectin) (Unigene77099_All) (Grunwald *et al.*, 2007), thionin (Unigene21824_All and Unigene43623_All) (Apel *et al.*, 1992) and jasmonate-induced genes (Unigene78255_All). Moreover, genes involved in defense responses that were also highly up-regulated in *eibi1* including *Lipoxygenase (LOX)* (Unigene72225_All) (Bell and Mullet, 1991; Yang *et al.*, 2012), heat-shock protein (Unigene78052_All) (Vierling, 1991), Cysteine-rich secretory protein family (Unigene68601_All) and RNase S-like protein (Unigene19875_All and Unigene66056_All) (Zheng *et al.*, 2014). But aspartyl protease family protein in insect digestion (Unigene78045_All) (Ryan 1990) and proteinase inhibitor I in response to wounding or insect attacks (Unigene57171_All) (Lee *et al.*, 1986) were down-regulated in *eibi1* .

**Table 3:** Relative expression of defense-related Unigenes.

Unigene ID	Related function	Log_2_ (*eibi1*_RPKM/23-19_RPKM)
Unigene77099_All	Jacalin-related lectin 31 (Horcolin)	13.55124
Unigene66257_All	WIR1A	12.09785
Unigene67107_All	Plant basic secretory protein (BSP) family protein	12.20341
Unigene78255_All	Jasmonate induced protein	10.92834
Unigene78697_All	Pathogenesis-related protein	10.70807
Unigene66056_All	RNase S-like protein (Ribonuclease T2 family)	10.60660
Unigene78238_All	Defense response to fungus (pathogenesis-related protein 4)	10.46467
Unigene78121_All	23kDa jasmonate-induced protein	9.708934
Unigene68601_All	Cysteine-rich secretory protein family (defense/immunity protein activity)	9.152529
Unigene78052_All	Hsp90 protein	8.668116
Unigene69289_All	Response to oxidative stress (Peroxidase)	8.310662
Unigene72225_All	Lipoxygenase (LOX)	3.441247
Unigene21824_All	Plant thionin	5.231692
Unigene43623_All	Plant thionin	4.445976
Unigene19875_All	RNase S-like protein (Ribonuclease T2 family)	3.851412
Unigene78082_All	Response to oxidative stress (Peroxidase)	3.699962
Unigene78045_All	Eukaryotic aspartyl protease family protein	-9.089449
Unigene57171_All	Response to wounding (Potato inhibitor É family)	-7.55606
Unigene33356_All	Response to stress (ABA/WDS induced protein)	-4.05753

Column named log_2_ (*eibi1*_RPKM/23-19_RPKM) indicates the expression of Unigenes in *eibi1* compared to 23-19. Plus (omitted): up-regulated, minus: down regulated.

Unigene69289_All and Unigene78082_All encoding peroxidase to defend plants against reactive oxygen species (ROS) (Wang *et al.*, 1999; Mittler, 2002) were clearly up-regulated in *eibi1*. Unigene33356_All involved in response to water deficit stress (WDS) mediated by ABA was down-regulated (Padmanabhan *et al.*, 1997) (Table 3). In addition, about four DEGs were associated with sugar transportation, seven DEGs were related to protein tyrosine kinase, eight DEGs were involved in cytochrome P450 and another nine DEGs were annotated as cellulose synthase (Table S7).

## Discussion

We carried out a transcriptome analysis of the *hvabcg31/eibi1* mutant and its isogenic wild barley 23-19 in the leaf elongation zone. About 78,799 Unigenes with average length of 645 bp were assembled and analyzed for gene expression in both mutant and wild type. A SNP filtering analysis between *eibi1* and 23-19 Unigenes identified a SNP between Unigene20340_23-19 and Unigene66600_*eibi1*, which were partial sequences of an ABCG transporter gene. Actually, this ABCG transporter gene was *Eibi1* and the SNP was the *eibi1* mutation (Chen *et al.*, 2011b). In the Morex *eibi1* (155F_2_) genetic map (Chen *et al.*, 2009), the six marker polymorphisms of all eight EST sequences were confirmed by the comparison of *eibi1* Unigenes and Morex contigs (http://webblast.ipk-gatersleben.de/barley). Two markers had polymorphisms in introns, so they could not be confirmed by RNA-Seq data. These results infer that RNA-Seq may be used for mutation identification and/or mutant genetic mapping, similar to SNP calling (Piskol *et al.*, 2013).

It is of interest that both studies failed to identify the most known differentially expressed cuticle genes. Using the second leaves of *eibi1* mutant and wild-type seedlings 10 days after germination at 22 °C in growth chamber for transcriptome analysis by a 22-k Barley1 Affymetrix microarray, similar results to the present study were found (Yang *et al.*, 2013). Only two Unigenes and one gene closely linked to cuticle showed differential expression in the present and previous study, respectively. Two *GDSL* Unigenes are close homologs of *cutin synthase-like2* (*CUS2*) in tomato (*Solanum lycopersicum*) (Yeats *et al.*, 2014). CUS protein is predicted to catalyze cutin polymerization and is located extracellularly. *HvABCG31/Eibi1* is assumed to function in transporting substrates for CUS. The reduced amount of cutin monomers transported out of the epidermal cells in *eibi1* mutant (Chen *et al.*, 2011b) may be responsible for the down-regulation of the two *GDSL* Unigenes. In the previous study, one cytochrome P450 monooxygenase gene (*CYP450*) homologous to *At4g39490* was up regulated. *At4g39490* is a paralog of *At1G57750* (*AtMAH1*) that is involved in the cutin monomer synthesis (Greer *et al.*, 2007). The plant cuticle is an extracellular lipid structure. The limited impact on lipid metabolic networks is also found in Arabidopsis (*Arabidopsis thaliana*) cuticle mutant *glossyhead1* (*gsd1*) (Lü *et al.*, 2011). The *eibi1* mutant showed only three lipid genes up-regulated and five lipid genes down-regulated, whereas the *gsd1* mutant exhibited three lipid genes up-regulated and eight lipid genes down-regulated (Table 2). The defective cuticle leads to a particularly severe water loss from *eibi1* mutant leaves. Thus, *eibi1* plants suffer from dehydration stress (Chen *et al.*, 2011b). *HvABCG31/Eibi1* was not differentially expressed in the *eibi1* mutant in both studies, indicating that the leaf phenotype may be responsible for differentially expressed stress- and defense-related genes. Similarly, the Arabidopsis cuticle mutant *gsd1* has a large effect on genes responsible for abiotic and biotic stress (Lü *et al.*, 2011).

*eibi1* mutant plants are unable to export cuticular lipids (mostly 16- and 18-carbon fatty acids) from the epidermis cells, leading to an accumulation of intracellular lipids (Chen *et al.*, 2011b). Both 16- and 18-carbon fatty acids are involved in defense to modulate basal, effector-triggered, and systemic immunity in plants (Kachroo and Kachroo, 2009). In a previous study, certain cuticle breakdown monomers were shown to function as elicitors of defense reactions in plants (Kolattukudy 1985; Schweizer *et al.*, 1994, 1996b). Treatment of cutin monomers can trigger alkalinization, higher ethylene (ET), and accumulation of defense-related genes in potato (Schweizer *et al.*, 1996a), and production of H_2_O_2_ in cucumber (Fauth *et al.*, 1998). Permeable *Arabidopsis* cuticle mutants (*pec/atabcg32*) and rice plants compromised in *OsABCG31* expression also showed increased resistance to *Botrytis cinerea* and *Magnaporthe oryzae*, respectively (Reina-Pinto and Yephremov, 2009; Bessire *et al.*, 2011; Garroum *et al.*, 2016;). In the study on the *osabcg31* knockout mutant, and hairpin RNA interference (RNAi)-down-regulated *OsABCG31* plant lines, genes involved in pathogen resistance were constitutively up-regulated. A link to increased expression of defense genes and dwarfism was also revealed (Garroum *et al.*, 2016). A growth reduction has been described as a typical trade-off for plant defenses (Bowling *et al.*, 1994; Yan *et al.*, 2007). In another study, cutinase-expressing plants (CUTE plants) displayed almost complete immunity toward *Botrytis cinerea*, although this was not found to correlate with the induction of genes coding for various pathogenesis-related (PR) proteins (Chassot *et al.*, 2007). It was also reported that cutin deficiency significantly affected the fruit response to biotic stress and significantly enhanced fruit sensitivity to the fungal post-harvest pathogen *Colletotrichum coccodes* (Shi *et al.*, 2013). However, not all mutants affected in the cuticle structure showed an enhanced resistance to certain pathogens. It was shown that only certain kinds of cutin monomers induced the expression of defense genes in tomato (Buxdorf *et al.*, 2014; Metraux *et al.*, 2014).

The use of an inducible defense system, named acquired resistance (AR) has been well demonstrated in plants (Heath, 2000; Conrath, 2006; Winter *et al.*, 2014). AR or systemic acquired resistance (SAR) is a form of inducible resistance that is triggered systemically in plants. The mechanism is associated with a systemic accumulation of salicylic acid (SA). Another potential lipid-derived AR signal is the oxylipin-derived defense hormone jasmonic acid (JA), which may be an early signal establishing systemic immunity (Vlot *et al.*, 2008). AR is also associated with the coordinated activation of genes encoding PR proteins (Walters *et al.*, 2014). How does the whole system respond after perceiving signals like these over-produced or over-accumulated cuticle monomers? It is very likely that JA or even other lipid signals play important roles in the generation and/or transmission of mobile signals for AR (Conrath, 2006), although it is also possible that SA initiates the process (Colebrook *et al.*, 2012).

The role played by ABA is intriguing. It has been shown that no significant change was found in the *eibi1* mutant (Chen *et al.*, 2004). Yet, ABA-related genes were differentially expressed in the mutant. It was suggested that ABA is likely to be involved in the suppression of wound-induced ROS, when plants are kept under dry conditions (Apel and Hirt, 2004). Several reports showed a link between ABA and increased susceptibility to pathogens that was mostly explained by antagonistic interactions of ABA with defense signaling controlled by SA, JA, or ET (Mauch-Mani and Mauch, 2005).

**Figure 6:**
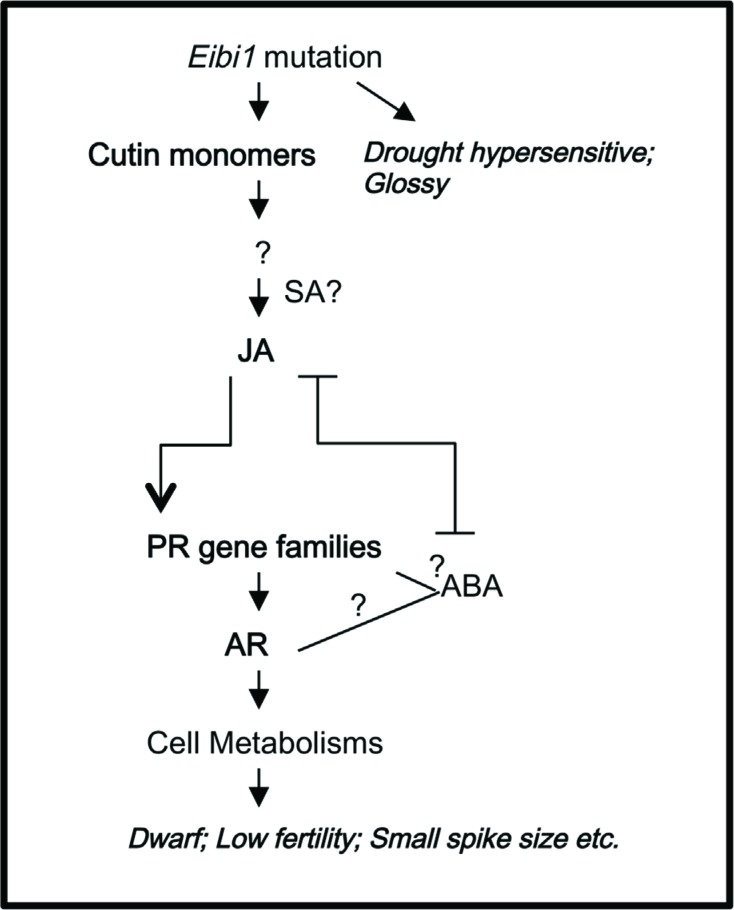
Possible acquired resistance (AR) pathway of *eibi1*.

Overall, this mutant could be a model of self-induced AR (Figure 6). The mutation of *eibi1* leads to an excessive amount of monomers or precursors of cutin. Then, possibly through unknown plant immune mechanisms, the plant reacts with increased JA, which further up-regulates PR gene families. This leads to a broad spectrum response, and a metabolic regulation possibly as compensatory process (Metraux *et al.*, 2014). Further applications on molecular mechanisms and agriculture should be studied.

## Supplementary Materials

The following online material is available for this article:

Table S1 - Quality of Unigenes.

Table S2 - Distribution of unique-mapped reads of the assembled unigenes of 23-19 and *eibi1*.

Table S3 - Functional classification by Gene Ontology (GO) analyses of all Unigenes.

Table S4 - Detailed SNP Statistics.

Table S5 - Polymorphisms were confirmed by *eibi1* Unigenes and Morex contigs.

Table S6 - List of DEGs between 23-19 *vs* *eibi1*.

Table S7 - DEG annotated by Pfam with GO terms.

Table S8 - DEGs within the top 20 up -and down-regulated genes that failed Pfam2go mapping.

Table S9 - Regulation of homologs of known cuticle genes.

Table S10 - Experimental validation using qRT-PCR and primer sequences for qRT-PCR expression analysis.
